# Application of Artificial Intelligence in the Detection and Differentiation of Colon Polyps: A Technical Review for Physicians

**DOI:** 10.3390/diagnostics9030099

**Published:** 2019-08-20

**Authors:** Wei-Lun Chao, Hanisha Manickavasagan, Somashekar G. Krishna

**Affiliations:** 1 Department of Computer Science, Cornell University, New York, NY 14853, USA; 2 Department of Computer Science and Engineering, Ohio State University, Columbus, OH 43210, USA; 3 Division of Gastroenterology, Hepatology and Nutrition, the Ohio State University Wexner Medical Center, Columbus, OH 43210, USA

**Keywords:** colonoscopy, colon polyp, artificial intelligence, computer-aided diagnosis, machine learning

## Abstract

Research in computer-aided diagnosis (CAD) and the application of artificial intelligence (AI) in the endoscopic evaluation of the gastrointestinal tract is novel. Since colonoscopy and detection of polyps can decrease the risk of colon cancer, it is recommended by multiple national and international societies. However, the procedure of colonoscopy is performed by humans where there are significant interoperator and interpatient variations, and hence, the risk of missing detection of adenomatous polyps. Early studies involving CAD and AI for the detection and differentiation of polyps show great promise. In this appraisal, we review existing scientific aspects of AI in CAD of colon polyps and discuss the pitfalls and future directions for advancing the science. This review addresses the technical intricacies in a manner that physicians can comprehend to promote a better understanding of this novel application.

## 1. Introduction

Colorectal cancer is the third most common cancer affecting both men and women in the United States and is the second leading cause of cancer-related deaths [1]. Fortunately, colorectal cancer incidence and mortality rates have been decreasing steadily over the last three decades in the United States, in part because of effective screening techniques [2]. The 1993 landmark National Polyp Study demonstrated that the incidence of colorectal cancer could be reduced by colonoscopy and polypectomy of adenomatous polyps [3], which has been confirmed in many follow-up studies [4,5,6,7]. Thus, many major societal guidelines have recommended colonoscopy as the preferred colon cancer screening strategy [8,9,10]. 

Adenoma detection rate (ADR)—the percentage of screening colonoscopies with at least one adenoma—has become a key quality measure in the United States for evaluating an endoscopist’s ability to find adenomas. Higher ADRs are associated with lower post-colonoscopy colorectal cancers and lower colorectal cancer mortality [9,11]. Although the ADR should ideally reflect adenoma prevalence, studies have demonstrated that ADR varies widely among endoscopists (7–53%) [11], and 20–30% of adenomas could be missed during screening colonoscopies [12,13]. Alarmingly, colorectal cancer had a miss-rate on screening colonoscopy of 4–5% [11,14,15], and 7.2–9% reported interval cancers or colon cancers that occur despite adherence to current guideline [2].

Several adjunct techniques and devices are under investigation for improving an endoscopist’s ability to detect adenomas. In order to increase efficiency and decrease overall healthcare costs, efforts have been underway to develop methods to accurately diagnose and disregard diminutive (<5 mm), non-neoplastic polyps and remove only precancerous polyps. One such method gaining significant traction is computer-aided diagnosis (CAD), which is a computer-assisted image analysis that incorporates both increased polyp identification and histopathologic differentiation without modifications to the colonoscope or the actual procedure. Furthermore, unlike alternative techniques (such as narrow-band imaging and virtual chromoendoscopy), CAD is largely operator-independent.

## 2. Computer-Aided Diagnosis

In recent years, artificial intelligence (AI) has made significant progress owing to the development of deep neural networks and machine learning algorithms, especially in the area of computer vision [16]. Convolutional neural networks (CNNs) are a class of deep neural networks that are highly effective at performing image and video analyses. CAD–CNN models for colonoscopy could assist endoscopists in detecting polyps and performing optical diagnosis [17,18,19]. These AI-based models have great potential for increasing the adenoma detection rate (ADR) and reducing the cost of polypectomy for hyperplastic polyps [18,20,21]. CNNs are trained using thousands of colonoscopy images to identify and differentiate between hyperplastic and adenomatous polyps [17,20,21,22,23]. In order to be maximally effective, the polyp identification module should have a high sensitivity for the detection of polyps, with low rate of false positives, while maintaining fast processing speeds to be applicable in near-real time during colonoscopy. 

Current versions of CAD models in research studies reveal a detection accuracy of 89–95% where 100–466 polyps per study have been delineated [22,24,25,26,27,28,29]. Several retrospective studies have demonstrated sensitivities higher than 90% [22,24,25,26,27,28,29], with a negative predictive value of 95–97% [27]. In a recent large study involving 8641 colonoscopy images containing 4088 polyps, a CAD–CNN system identified polyps with an accuracy of 96.4% and an area under the receiver operating characteristic curve of 0.991 [23]. In another recent prospective study of 1058 patients who were randomized to colonoscopy with and without CAD, ADR (29.1 vs. 20.3%, *p* > 0.001), and the mean number of adenomas per patient (0.53 vs. 0.31, *p* < 0.001) were significantly higher for the CAD group [30]. Both studies further demonstrate strong evidence that CAD, by detecting more polyps, may be able to improve ADR and lower adenoma miss rates, while limiting unnecessary polypectomies of non-neoplastic polyps.

In the application of AI for colonoscopy, automatic detection and characterization of colorectal polyps has attracted the most attention compared to the differentiation of polyps as adenomatous or hyperplastic. The former aims to detect polyps, irrespective of their pathology (neoplastic polyps or hyperplastic polyps). The later then helps to visually classify the detected polyps into pathological categories. In the literature, polyp detection is mostly studied using white-light imaging, while polyp classification requires more advanced imaging such as magnifying narrow-band imaging [21].

## 3. Technological Developments in Artificial Intelligence 

Data-driven approaches based on machine learning have been a major focus for modern developments in AI. Considering image classification as an illustrative example, in order to build a machine learning model, labeled training data (images annotated with corresponding class names) are collected, and used to optimize the model by evaluating performance on the training data. Early methods usually divided the model into two parts, (i) feature extraction, and (ii) classification, in which the former extracts essential features of an image and the later classifies the image according to the extracted features. Machine learning algorithms can learn the classification part, but the feature extraction part often requires human efforts of engineering [31].

Recently, deep learning, which involves modeling the two (feature extraction and classification parts) jointly by deep neural networks (DNNs) and learning the entire model using machine learning algorithms, has attracted significant attention due to its efficient performance in various applications [16]. Deep learning bypasses human engineering by learning more powerful features directly from the training data. For the image and video domains, CNNs are the most popular class of DNNs. CNNs perform layers of convolutions with translation-invariant filters, inherently modeling the property that an object (such as polyps) may appear in various locations in an image [32]. 

As we expect to generalize the learned model to images beyond training data, preventing “overfitting,” a phenomenon that a machine performs well on training data but poorly on other test data, is a significant challenge in machine learning. Overfitting occurs when a learning machine is overly tuned to the data it was trained upon. Two common strategies to prevent overfitting are to collect more training data and limit the models’ complexity [33]—with more training data, the learned model is likely to capture more generalizable knowledge, and limiting the complexity prevents the model from overly spending its capacity to fit the training data. Ideally, we would like to collect more data, but it can be laborious, costly, and resource intensive. In contrast, limiting the complexity is more efficient but may sometimes lead to “underfitting,” a phenomenon that a machine cannot perform well even on the training data. Choosing the appropriate strategy is thus dependent on the machine learning problem at hand, usually determined by (cross) validation: keeping a portion of data unused for training and using it to validate the model’s performance. These two strategies, however, are not mutually exclusive and can be applied simultaneously [33]. When applying AI technology, especially deep learning approaches whose models (neural networks) are usually highly complex and data-hungry, we must take generalizability and overfitting into account in learning the model since the goal is to perform CAD during colonoscopies on actual patients (unseen in training) with unique endoscopic characteristics.

## 4. Technical Aspects of Detecting Polyps during Colonoscopy

While early works on polyp detection were mostly built upon the pipeline beginning with hand-crafted (derived variables/covariates) features followed by classification at different regions of an image, recent applications of deep learning for polyps’ detection have mostly replaced these modalities [34,35]. Current studies consider spatial features of the polyps where CNNs are applied to individual frames (or images) of the colonoscopy videos [18].

A color image of a colonoscopy frame capture with a polyp can be represented by a three-dimensional (3D) tensor: the first two dimensions are along the image width and height, while the third dimension is along the color channels. In this case, existing works apply two-dimensional (2D)-CNNs, where the convolutions are performed along the width and height dimensions, and the filters are taught to capture the spatial patterns of polyps along these two dimensions. Moreover, since detection involves indicating the presence of polyps and localizing them, the output of the CNNs should also contain both data. To this end, Urban et al. designed multiple CNN architectures to perform binary classification of the presence or absence of, and regress the bounding box location of polyps on each frame [23]. Zhang et al. modified the single shot multibox detector (SSD), a CNN model initially proposed for common object detection, to detect polyps [23,36]. Shin et al. further applied a more sophisticated object detector, faster region Region(R)-CNNs, which is commonly known as a two-stage detector [37]. One-stage sensors, such as SSD, operate much faster but have lower detection accuracy than two-stage sensors, such as faster R-CNNs. Nevertheless, recent work by Lin et al. showed that, with a properly designed learning objective, single-stage detectors could achieve comparable performance to two-stage sensors without losing the advantage in running time [38]. Instead of localizing the polyps by regressing the bounding box locations, Wang et al. applied fully convolutional networks that were initially proposed for image semantic segmentation. Fully convolutional networks can directly predict if each image "pixel” belongs to polyps [30]. 

Some recent studies have the temporal information in colonoscopy videos as additional cues for the accurate detection of polyps. Tajbakhsh et al. proposed a two-step approach, which begins with detecting candidate regions of polyps using geometric features like shapes and sizes. Subsequently, the model learns multiple 2D-CNNs to extract features around each region, including one 2D-CNN that concatenates the preceding and successive frames along the color dimension to learning spatial-temporal patterns of polyps [39]. Yu et al. and Misawa et al. applied 3D-CNN, a more suitable model for video data, to learn the spatial-temporal patterns. A video can be represented by a four-dimensional (4D) tensor in which the fourth dimension corresponds to time. A 3D-CNN then performs convolutions along the width, height, and temporal dimensions [40,41]. To accommodate the significant variation in the morphology of the colon polyps, Yu et al. and Shin et al. applied the learned model in real-time during colonoscopy, and then used the recorded video together with the initial detection results to further adjust (fine-tune) the model. The modified model was then re-applied to the original video for the final detection [37,41]. 

## 5. Technical Aspects of Classifying Colon Polyps

Most studies in the current literature have applied a binary classification in differentiating colonic polyps as either hyperplastic or adenomatous lesions. For polyp detection, the models are trained to distinguish between polyp regions and nonpolyp regions. However, for accurate polyp classification, the models are trained to differentiate between hyperplastic versus adenomatous polyps, which requires the extraction of more granular features. Since the information on the differentiation of polyps from white-light endoscopy is insufficient, most polyp classification CAD models exploit more advanced imaging such as narrow band imaging (NBI) [21].

Early studies on the differentiation of colonic polyps extracted features of the lesion. For example, Tischendorf et al. and Gross et al. obtained data on blood vessel features from magnified NBI images, while Misawa et al. and Mori et al. extracted textural features by analyzing the contrast difference of pixels [22,26,42,43]. More recent studies have applied DNNs for extracting more discriminative features. Byrne et al. used CNNs to each image frame to classify polyps into specific categories. When analyzing the colonoscopy video, the investigators then accumulated credibility scores by examining how the classification of polyps fluctuates across successive frames [27]. Ribeiro et al. also utilized CNNs, but to image patches and increase the size of training data [44]. These two studies trained CNNs from the beginning using colonoscopy images annotated with pathology categories. Another two studies by Zhang et al. and Chen et al. applied pre-trained CNN models using natural images and fine-tuned the final layer of the classifier on the labeled colonoscopy images, a useful strategy to leverage more images for learning powerful features [29,36].

## 6. Technical Aspects of Combined Detection and Classification of Colon Polyps

At least two studies have proposed CAD models that detect and then classify colon polyps [36,45]. CAD models for the detection of polyps can be potentially equipped with the functionality of polyp classification with some modifications. For example, faster R-CNNs applied by Shin et al. first propose candidate regions that might contain polyps and then classify each region into polyps or not (a binary classification problem) [37]. By modifying the binary classification problems into a three-class classification problem: (i) hyperplastic, (ii) adenomatous, or (iii) not a polyp, a faster R-CNN model could simultaneously perform polyp detection and classification.

## 7. Future Directions for the Detection and Classification of Colon Polyps

To date, the procedure of colonoscopy and detection of polyps remains highly operator-dependent with significant interoperator variability. The overall pooled miss rate in a meta-analysis is 22% for polyps of any size [46]. Multiple investigators are developing CAD systems for real-time polyp detection during colonoscopy. Some CAD systems can also provide “optical biopsies” in differentiating the type of colon polyp. However, before implementing CAD into routine clinical practice or guidelines, regulatory impediments that need to be addressed include the complexity of deep-learning guided clinical decision-making which is frequently contemplated as a “black box”. However, we would like to look forward to a future “human–computer collaboration” that will provide the highest quality colonoscopy for all patients.

While AI technology, especially deep learning, has been used in recent studies to improve CAD models for the detection of polyps during colonoscopy, several challenges remain. First, most recent studies are based on pre-recorded videos or image frames, and not on real-time video feeds. Second, many studies have utilized a limited number of colon polyps for both the training and evaluation of CAD models, compared to studies on natural images [38]. Third, simple coarse-grained categories have been utilized for polyp detection (presence versus absence of colon polyps) and classification of polyps (hyperplastic or adenomatous) [17,21,22]. 

Modern AI technology has led to remarkable progress in the field of biomedical engineering, but its effectiveness will likely be limited by the machine’s ability to explain its decisions to users, especially in medical and healthcare applications. Hence, interpretable AI has evolved. Specifically, for the detection of polyps during colonoscopy, one promising direction is to develop an AI-based CAD system that can explain its decisions using the classification rules developed to help endoscopists differentiate polyps Narrow-band imaging International Colorectal Endoscopic (NICE) and Simplified Identification Method for Polyp Labeling during Endoscopy (SIMPLE) classification) [20]. In this way, endoscopists can easily understand the CAD system’s decisions. Another issue involves “fairness”, where human data train the CAD models. Any bias from the training data can be transferred to the CAD model leading to skewed data that does not represent the entire population. For example, the training data could have disproportionately higher men or women, or patients from just one specific race, and thus the decision of the CAD model will not represent a “normal” population. 

One further issue is the human–machine collaboration. To this end, explainable AI will play a key role in enabling human–machine communication. Moreover, instead of learning and evaluating CAD models using average performance (for example, average ADRs), we should include and emphasize colonoscopy images and videos with missed polyps by the endoscopists. These cases are where CAD models can benefit the most, but considering average performance will likely encourage the models to solve common and less complicated cases. We must develop novel evaluation metrics and model training strategies to emphasize such challenging cases to maximize the benefit of CAD in human–machine collaboration. Finally, it is well known that data-driven models suffer from data discrepancy between training and testing. This occurs when the training data are statistically very different from the future testing data due to patients’ attributes, equipment, hospitals, or even the data annotation quality. To scale up and widen the applicability of CAD for colonoscopy, training data must be comprehensive and include patients from different demographic areas, multiple centers, and endoscopists with varying levels of experience.

In conclusion, CAD and AI techniques such as deep learning permit expedited processing of large amounts of image data and can potentially assist clinicians in decision making, such as detection and classification of colonic polyps. The number of applications utilizing AI will expand over the next few years with improved computational power and complex decision algorithms. Avoiding the various biases in training data is critical for establishing the accurate CAD model.
